# Complete genome sequence of *Lactobacillus acidophilus* LA120 from the NORDBIOTIC collection

**DOI:** 10.1128/mra.00636-26

**Published:** 2026-07-30

**Authors:** Agnieszka Szczepankowska, Bożena Cukrowska, Tamara Aleksandrzak-Piekarczyk

**Affiliations:** 1 Institute of Biochemistry and Biophysics, Polish Academy of Sciences (IBB PAS) 90863, Warsaw, Poland; 2 Department of Research and Development, Nordic Biotic Ltd., Warsaw, Poland; Portland State University, Portland, Oregon, USA

**Keywords:** *Lactobacillus acidophilus*, complete genome, probiotic, hybrid assembly, Oxford Nanopore Technologies

## Abstract

The complete genome sequence of *Lactobacillus acidophilus* LA120, from the NORDBIOTIC collection, was generated using Illumina and Oxford Nanopore sequencing. The genome comprises a circular 1,991,955-bp chromosome with 34.5% G + C content, providing a reference for comparative genomic and functional analyses of this strain.

## ANNOUNCEMENT

*Lactobacillus acidophilus* is a lactic acid-producing, gram-positive species whose strains are widely used as starter bacteria in dairy applications and as probiotics (1). *L. acidophilus* LA120 is part of the NORDBIOTIC collection of Nordic Biotic Ltd. (Warsaw, Poland) and is deposited at the Leibniz Institute DSMZ-German Collection of Microorganisms and Cell Cultures GmbH as DSM 33,795. A multi-strain probiotic formulation containing LA120 has been shown in clinical trials to reduce respiratory symptom severity and improve symptoms of diarrhea-predominant irritable bowel syndrome (2, 3), and LA120 has been associated with a vitamin B3-related metabolomic profile, including elevated proline and succinate levels, relevant to the gut-skin axis (4). To facilitate genomic exploration of this strain, we performed whole-genome sequencing.

LA120 was supplied from the NORDBIOTIC collection as a freeze-dried powder, revived in de Man, Rogosa, and Sharpe (MRS; Oxoid) broth, plated on MRS agar (Oxoid), and incubated overnight at 37°C under anaerobic conditions. Genomic DNA was isolated from an overnight culture grown under the same conditions. Cells were collected by centrifugation and pretreated with lysozyme (20 mg/mL; Sigma-Aldrich) and mutanolysin (5 U/mL; A&A Biotechnology) for 15 min at 37°C, followed by sodium dodecyl sulfate lysis, proteinase K digestion, and organic extraction as previously described (5).

DNA was sequenced using a hybrid approach combining short-read Illumina MiSeq sequencing and long-read Oxford Nanopore Technologies (ONT) sequencing. Illumina libraries were constructed using the NEBNext Ultra II FS Kit (New England Biolabs) and sequenced on an Illumina MiSeq platform (2 × 300 bp). Raw reads were inspected with FastQC v0.11 and filtered with fastp v0.23.4 (6), resulting in 3,739,840 reads and 1,105,397,352 nt of sequence.

ONT libraries were constructed using the Native Barcoding Kit V14 and sequenced on a P2 Solo instrument with an R10.4.1 flow cell, all from Oxford Nanopore Technologies. Raw signals were base called using Dorado (ONT). Adapters were removed with Porechop_ABI (7), reads were quality-filtered with Chopper v0.12.0b, and read metrics were assessed with NanoPlot (8), yielding 40,325 reads and 408,777,464 nt of sequence (~205 × coverage), with an average length of 10,137.1 nt and an N50 of 17,206 nt. Assembly and circularization of ONT reads were performed using Autocycler v0.5.2 (9), supported by multiple assemblers: Flye v2.9, Canu v2.3, Raven v1.8.1, Miniasm v0.3-r179, metaMDBG v1.2, NECAT v0.0.1, and NextDenovo v2.5.2 (10–16). The assembly was polished with Illumina reads using PyPOLCA v0.4.0 and Polypolish v0.6.0 (17, 18). All software was used with default parameters. The final sequence was rotated relative to *dnaA* using dnaapler v1.3.0 (19). QUAST v5.0.2 (20) identified a single-contig assembly of 1,991,955 bp with 34.5% G + C content and no gaps (N ratio = 0.0), while analysis of the assembly graph file in GFA format in Bandage v0.9 (21) confirmed a circular, non-branching genome structure. The National Center for Biotechnology Information Prokaryotic Genome Annotation Pipeline v6.10 was used for genome annotation (22), predicting 1,851 coding sequences (CDSs), including 1,794 protein-coding CDSs and 57 pseudogenes, as well as 61 transfer RNAs, 12 ribosomal RNAs, 3 non-coding RNAs, and 1 CRISPR array. The finalized genome of LA120 provides a basis for detailed analyses in subsequent studies.

## Data Availability

The whole-genome shotgun project has been deposited in GenBank under accession no. JBWDMU000000000.1. The complete chromosome sequence described in this paper is available under accession no. JBWDMU010000001.1. The associated project and sample are available under BioProject accession no. PRJNA1437409 and BioSample accession no. SAMN56509523, respectively. Raw sequencing reads have been deposited in the Sequence Read Archive under accession nos. SRR37693798 (Illumina) and SRR37693792 (Oxford Nanopore Technologies).
